# Pressure knapping west of the Rhine during the Mesolithic? New evidence from Kerkhove (Belgium)

**DOI:** 10.1371/journal.pone.0330662

**Published:** 2025-09-24

**Authors:** Hans Vandendriessche, Colas Guéret

**Affiliations:** 1 Prehistory Research Unit, Department of Archaeology, Ghent University, Ghent, Belgium; 2 CNRS, UMR 8068 Technologie et Ethnologie des Mondes PréhistoriqueS (TEMPS), MSH Mondes, Nanterre, France; Universita degli Studi di Ferrara, ITALY

## Abstract

Until now, evidence for the use of pressure knapping in NW Europe during the Mesolithic has remained very scarce. In this paper, we present the technological (and functional) analysis of a new pressure knapped microbladelet assemblage from the Belgian site of Kerkhove. Attributed to the Middle Mesolithic (between 9525 and 8224 cal. BP), it marks an unexpected early appearance of this technique in the region, that strongly suggests knowledge transmission and contacts with the Maglemosian cultural area from Northern Germany and Southern Scandinavia where this technique was already present at that time. Nevertheless, based on the absolute scarcity of the evidence so far in NW Europe and based on the lack of genetic evidence, we argue that the spread of the pressure knapping technique to NW Europe did not involve large-scale demic diffusion as it was the case with its dispersal into Scandinavia. In Kerkhove, in addition, the limited size of the assemblage and the lack of other tools, cores and knapping waste related to pressure knapping and the lack of refits among the pressure knapped bladelets, indicates that it could have fulfilled a complementary role to the other lithic productions at the site, perhaps related to the (re)tooling of a very specific tool type akin to the slotted bone points/daggers known from the Maglemosian/Kongemosian area and the Baltic region.

## Introduction

**Fig 1:**
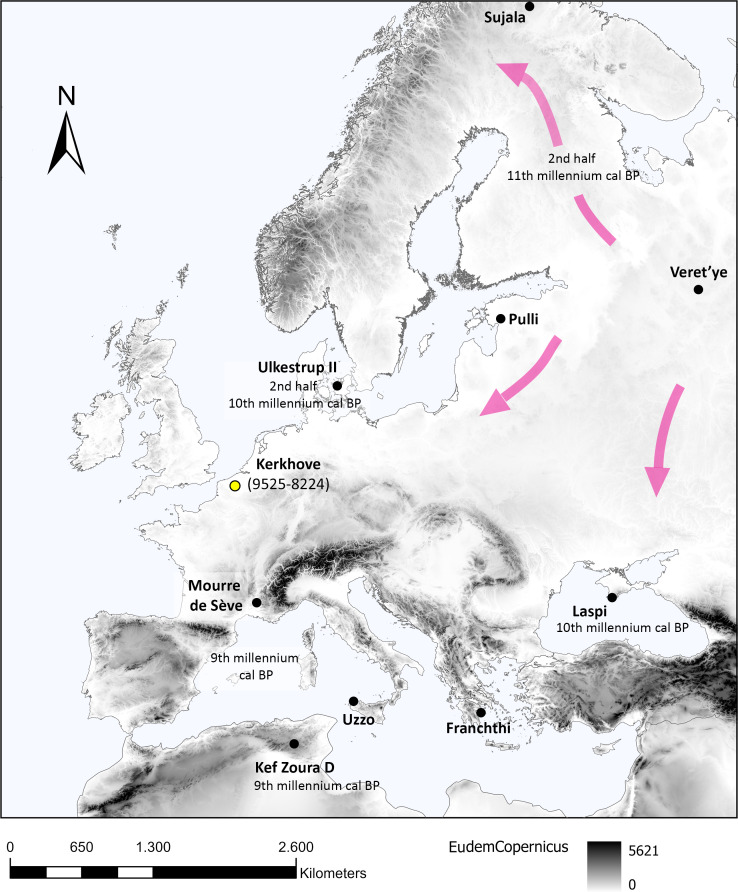
Possible diffusion routes of pressure knapping in Western Eurasia with important sites and approximate dates of the earliest appearance of pressure bladelets per region (data source for the DEM, https://doi.org/10.5270/ESA-c5d3d65).

The systematic use of pressure knapping to produce regular bladelets developed around ca. 20 000 BP in Palaeolithic societies in the area of Northern China, Mongolia and Southern Siberia [1]. From this center of invention, it gradually spread westward reaching Western Russia, the Baltic States, and Scandinavia during the Early Holocene (in the course of the 12^th^ – 10^th^ millennium cal BP [2,3]). In these areas, pressure knapped bladelets and cores are initially attested in Mesolithic assemblages belonging respectively to the Post-Swiderian cultural complex (i.e., Kunda, Butovo and Northern Scandinavian sites) and the Late Maglemose culture. A similar westward expansion likely also resulted in the emergence of pressure knapping associated with the appearance of trapezes in the Black Sea area (Southern Ukraine and Moldova [4]) and slightly later around the Mediterranean basin [1], e.g., in the Upper Capsian in Northern Africa, as well as in the Late Mesolithic of Italy and Southern France during the 9^th^ millennium cal. BP [1,5–7] (Fig 1).

Along the Atlantic coast of Europe, more specifically in Northern and Western France, the Benelux and Western Germany, regular bladelets appear from the Late Mesolithic onward, linked to the so-called Montbani knapping style [8]. However, they are generally acknowledged to have been produced by indirect percussion [9–11]. Evidence for the use of pressure knapping is by contrast very scarce (limited to a handful of cores found in problematic contexts, see infra) and it remains unclear for the moment how and to what extent pressure knapping was further diffused into NW Europe.

In this paper, we would like to shed more light on this matter by presenting a context with regular microbladelet productions from the Belgian site of Kerkhove: C12, a Middle Mesolithic artefact cluster in which these microbladelets are found associated with small backed bladelets (so-called “rods” or l*amelles étroites à bord abattu*) and invasively retouched points.

Subsequently, comparing the results obtained on this artefact cluster with data from the wider European context, an attempt will be made to discuss the timing of the appearance of this technique in Northwestern Europe during the Mesolithic, as well as the nature of its application.

## Materials and methods

### Site presentation

**Fig 2:**
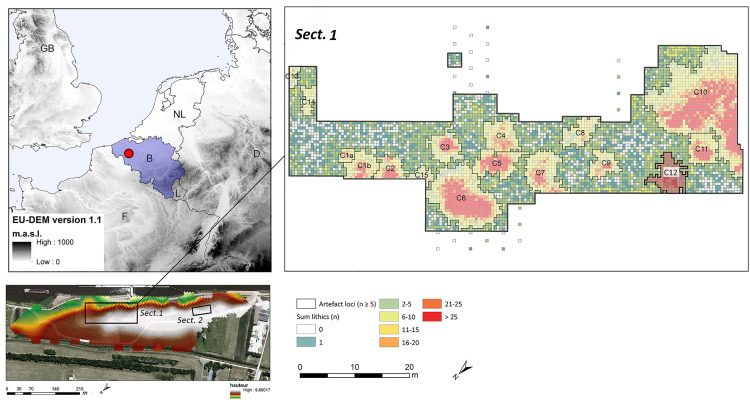
Geographic setting of Kerkhove, position of the two excavation trenches (Sect. 1 and Sect. 2) and spatial distribution of the seventeen artefact loci discovered at the site, including C12 (highlighted) (data source for the DEM, https://doi.org/10.5270/ESA-c5d3d65).

The site of Kerkhove is an extensive open-air site situated in NW Belgium, in the floodplain of the river Scheldt. In 2015–2016, seventeen spatially distinct artefact clusters were discovered there, on the slopes and the top of a NW-SE oriented alluvial levee (length>550 m, mean width ca. 80 m, mean height 3 m). The archaeological contexts owe their excellent preservation to the fact that this levee was sealed by peat formation from the second half of the 10^th^ millennium cal BP onwards and covered by alluvial clays during historic periods. On a regional scale, the site is of particular importance because of its rich faunal assemblage [12] and the high-resolution paleo-environmental records it yielded [13–15]. Moreover, it is also noteworthy because it was occupied approximately from the first half of the 11^th^ millennium cal BP to the 8^th^ millennium cal BP, therefore offering a unique long-term perspective on the Mesolithic occupation of Northern Belgium [16]. Nonetheless, it needs to be emphasized that the vast majority of artefact clusters (sixteen) at Kerkhove are associated with an Early to Middle Mesolithic occupation of the site and that only one artefact cluster, located higher on the levee in excavation trench 2, dates to the Late Mesolithic. While previous research entirely focused on the lithic technology, functional characteristics and spatial organization of the Early and Middle Mesolithic settlements adopting a more generalized perspective [17–19], as mentioned above, this paper specifically concerns one of the Middle Mesolithic artefact clusters, C12, that had not been studied in detail before (Fig 2).

### C12 and the Rhine-Meuse-Scheldt (RMS) Mesolithic

**Fig 3:**
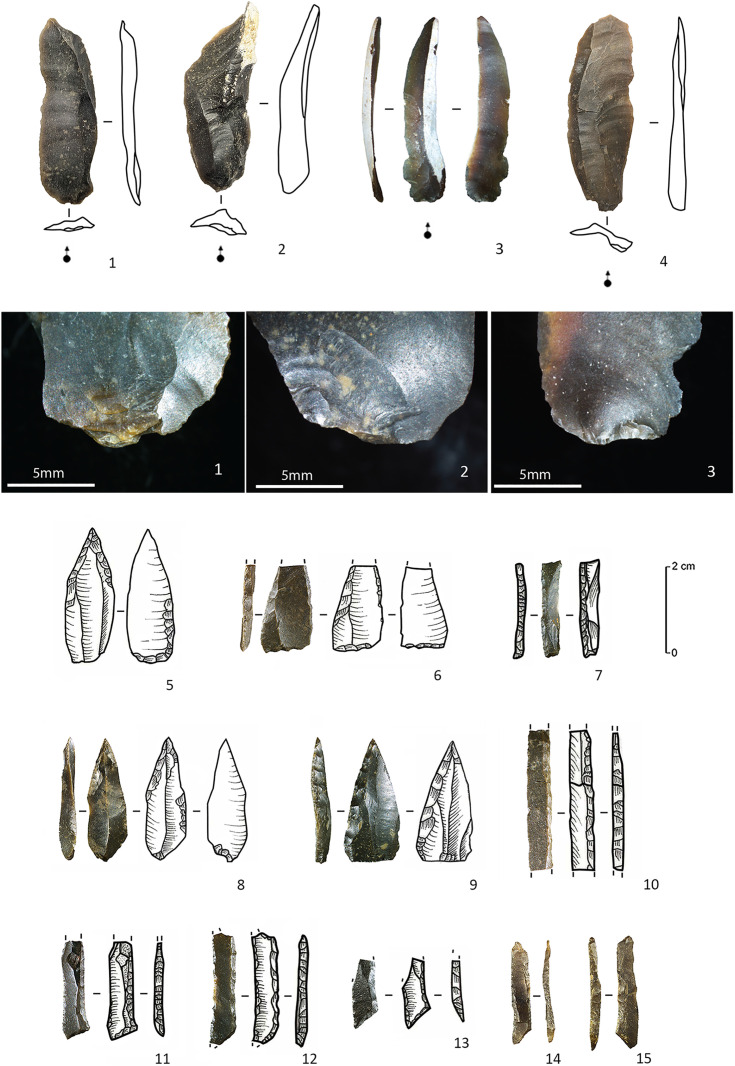
C12 – Direct percussion bladelets (1-4) with detailed view on the ventral proximal ends of bladelets 1-3, points with rounded or oblique base (5-6, 8), backed point (9), small backed bladelets (7, 10-15), among which four specimens with oblique truncation(s) (11-15).

C12 is situated at the western edge of the first and largest excavation area of the site. With just 1559 lithic artefacts (chips and debris smaller than 1 cm included, Table 1) found on a surface of 33 m^2^, it is among the smaller and lowest-density artefact clusters in Kerkhove. Microliths dominate the retouched toolkit (n = 59 or 96,72%), that is otherwise made up of a truncated bladelet and a burin. The vast majority of microliths in C12 (Fig 3; Table 2) are small backed bladelet fragments. Several of these feature one or two truncations in addition to their backed edge. When obliquely truncated, they often resemble narrow scalene triangles, however, without clear apex opposite to the truncation (n = 8). These small backed bladelets are accompanied by a mixture of invasively retouched points (among which a remarkable unfinished specimen made on a wide flake blank) and several other microlith types (i.e., a point with retouched base, three backed points and a scalene triangle, see Table 2).

**Table 1:** General typology of C12. * Only retouched artefacts were typologically considered as “tools”, even if many unretouched blanks were used, as demonstrated by the microwear research. **Changes in this table compared to Vandendriessche, 2022 are due to the new determinations made during the refitting and microwear research.

	N	%	N microwear
Cores	7	0,45	–
Preparation/rejuvenation	10	0,64	–
Flakes	189	12,12	8
Blade(let)s	251	16,10	18
Undet. flaking fragments	55	3,53	–
Artefacts < 1 cm	959	61,51	–
Debris	9	0,58	–
Tools*	61	3,91	1
Microburins	2	0,13	–
Burin spalls	1	0,06	–
Frost flakes	15	0,97	–
Total	1559	100	27

**Table 2:** Tool typology of C12. Changes in the tool spectrum compared to the counts that were published in Vandendriessche, 2022 are due to the new determinations made thanks to the refitting and microwear research.

	N	%
**Common tools**		
Retouched blade(let)	1	1,64
Burin	1	1,64
**Armatures**		
Small backed bladelet	20	32,79
Small backed bladelet with truncation	15	24,59
Point with invasive retouch	6	9,84
Triangle	2	3,28
Backed point	3	4,92
Point with retouched base	1	1,64
Undet. microlith fragment	12	19,67
Total	61	100,00

**Fig 4:**
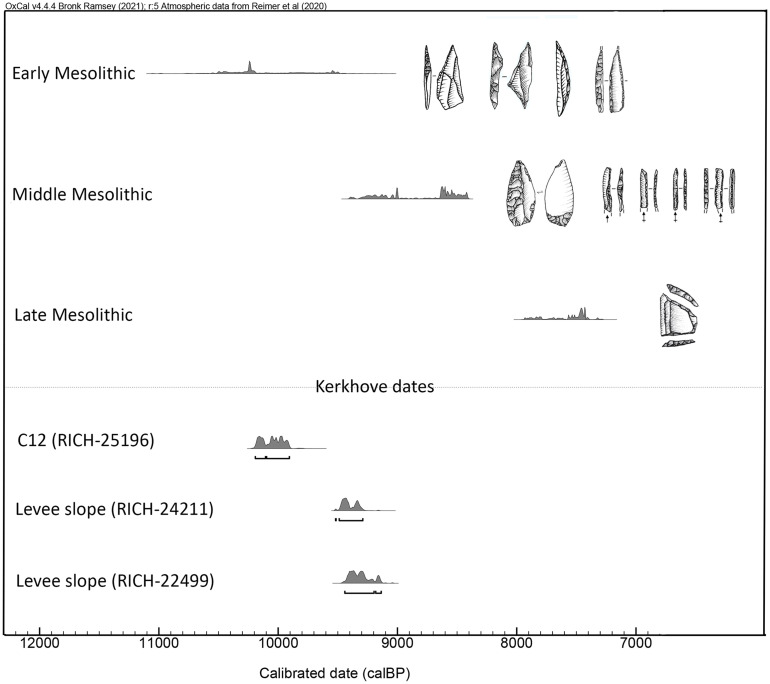
^14^C date plot with summed distributions of the Early Mesolithic, Middle and Late Mesolithic dates from the Rhine-Meuse-Scheldt region, adapted from Crombé [23] and dates mentioned in the text that are relevant for the dating of C12.

This combination of small backed bladelets and points with invasive retouch is typical for the Middle Mesolithic of the area and for assemblages belonging to the Rhine-Meuse-Scheldt (RMS) culture, *sensu* [20,21] (or to the Sonnisse Heide/Gelderhorsten assemblage type depending on the nomenclature that is followed [22,23]). RMS assemblages only occur along the southern North Sea basin, in the region more or less delimited by the rivers Seine in the south, Rhine in the north and the upper reaches of the Meuse and Moselle in the east. Chronologically, they are situated between ca. 9525 cal BP and 8224 cal BP based on the most recent analyses of the available ^14^C data [23,24]. Furthermore, from a typological point of view, they are clearly distinct from the preceding Early Mesolithic assemblages (characterized by different types of backed and truncated microliths, i.e., backed points, obliquely truncated points, points with retouched base, crescents, scalene triangles) and from the subsequent Late Mesolithic assemblages (characterized by trapezes, Fig 4).

The absolute dominance of small backed bladelets as well as the occurrence of invasively retouched points clearly demonstrates that C12 is primarily a Middle Mesolithic artefact cluster and that it was occupied broadly within the above-cited timeframe. However, the only absolute date directly associated with the cluster, carried out on a charred hazelnut shell, dates to 8921 ± 36 BP or 10192 to 9906 cal BP, Table 3). It is far too old for an RMS-assemblage (five to six centuries older than other Middle Mesolithic sites). Instead it probably refers to the ubiquitous Early Mesolithic occupation of the site in which hazelnuts were frequently exploited [18,25].

**Table 3:** ^14^C-dates mentioned in the text.

Context	Lab-code	Sample	BP date	95,4% prob. cal BP
C12	RICH-25196	*Corylus* shell (charred)	8921 ± 36	10192-9906
Levee slope (TR1 – unit VII)	RICH-24211	Bone (*Radius Bos Primigenius*)	8383 ± 39	9519-9291
Levee slope (TR3 – unit VII)	RICH-22499	Bone (*Cervid scapula*)	8304 ± 40	9441-9136

Faunal remains occur in all seventeen artefact clusters documented at the site. Unfortunately, they could not be dated due to poor- quality collagen or insufficient collagen yields. Only the faunal remains found on the slopes of the levee were better preserved and could be dated, thanks to their lower position in the landscape and the fact that they were sealed more quickly by peat formation. Two of the bones recovered from these slope sediments date to the start of the Middle Mesolithic (Table 3, RICH-24211 and RICH-22499) and although not directly associated with C12, they could offer a more reliable age estimate for the artefact cluster.

### Methods

C12 was subjected to a spatial analysis, to a lithic attribute analysis and refitting, as well as to an exhaustive microwear analysis. The attribute analysis entailed recording metrical, morphological andraw material attributes, as well as attributes related to knapping on each artefact larger than 1 cm, which provided quantitative data to support our technological interpretations. Raw materials were macroscopically assessed and compared to geological and archaeological samples available in regional databases. The refitting consisted of piecing together lithic artefacts in an attempt to reconstruct the original production sequences carried out by the prehistoric hunter-gatherers [26–30]. In doing so, it allows to gain highly detailed information on the knapping methods that were applied. Other than that, it also generates spatial information, visualized as refit lines, that can be used to understand artefact movements and/or the impact of taphonomic processes on the site [28,31–33].

All the artefacts of C12 were included in the refitting attempt, i.e., without making use of specific size cut-offs. Refits were recorded according to the chronological direction of the knapping sequence and by including only proximal fragments in the main sequence line [28,29]. Mapping and analysis of the artefacts and refit lines was carried out in ArcMap (Esri^tm^).

The use-wear analysis equally concerned all the lithic artefacts from C12 and consisted of screening the assemblage with a stereomicroscope (magnifications x5-x35) to identify the retouched tools and the unretouched blanks with macroscopic use traces. This resulted in an identification of the movements carried out with the implements and a first assessment of the relative hardness of the involved contact materials. Analysis under a metallographic microscope (x50-x200), to identify specific contact materials, was however not carried out. Although the main objective of this paper is not to detail the results of this use-wear approach, some of the results will be briefly addressed while discussing the functional purposes of specific knapping sequences, such as those related to pressure knapping (see *infra*).

## Results

### The direct percussion *Chaîne Opératoire*

234 bladelets and bladelet fragments were discovered in C12. The vast majority (n = 198) are direct percussion bladelets. They are characterized by their overall small dimensions (mean width of 9 mm and thickness of 3 mm), triangular sections, straight profiles and perhaps most typically by their irregular, converging or diverging outlines and dorsal ridges. The complete specimens and proximal fragments most commonly display pointed or linear butts (63%) combined with a high percentage of overhang abrasion(41%). Finally, ripples are generally speaking pronounced and proximal scars (‘*esquillements du bulbe*’, cf. [34]) occur relatively frequently (7%). Based on these attributes, it seems that they were most likely produced by direct percussion with a soft stone hammer, applied in a tangential manner and close to the striking platform edge [34–36]. While these bladelets constitute the main knapping objectives (used as unretouched blanks or further modified into specific tool types), C12 also yielded preparation elements (i.e., artefacts related to core-shaping), cores and retouched tools that belong to the same *Chaîne Opératoire*. The seven cores for example indicate that these bladelets were mainly detached in a semi-peripheral manner from opposed platform cores with relatively sharply inclined striking platforms (ca. 70° to 80°). The occurrence of crested bladelets/flakes implies a general preparation of the original volumes prior to bladelet debitage.

Several tool types also belong to this direct percussion Chaîne opératoire. This seems to have been the case for several microliths, such as the invasively retouched points, backed points, triangles and points with retouched base. Whether this includes the many small backed bladelets found in C12 is harder to determine with certainty, because of their limited size and high fragmentation rates. However, at least two small backed bladelet fragments display features that fit perfectly with the above descriptions (in one case a linear butt, in the second case very pronounced ripples on its ventral face). Other fragments show relatively pronounced curvatures that could also match with the idea that this tool category could have been a part of the direct percussion *chaîne opératoire* as well.

Other retouched tools are by contrast absent in C12, with the exception of a truncated bladelet and a burin. Nonetheless, an exhaustive screening under the stereomicroscope allowed to identify a significant number of unretouched bladelets and flakes with use-related edge-damage, i.e., 18 irregular bladelets and 8 small/elongated flakes, to which an additional eight blanks can be added with possible use-wear traces. All of the artefacts with use-wear traces are moreover between 2 cm and 5 cm long. From a functional point of view, the use-wear traces are not very diversified. Based on a preliminary analysis, most seem to be the result of cutting soft/moderately hard materials (20 use zones/UZs: butchery, hide?) and to a lesser extent of scraping hard (5 UZs: osseous materials/antler?) to semi-hard materials (7 UZs: wood, rigid plants, other…?).

Finally, the main flint varieties used for these productions (Table 4) are Upper Turonian flint from the Lille/Tournai area (UTLT) and a fine-grained translucent flint variety similar to the northern French Senonian flints. Upper Turonian flint from the Mons Basin (UTMB) and a flint nodule with a heavily rolled cortex (fluviatile pebble) were also worked in C12, but to a lesser extent [18,37]. Lastly, eight artefacts, exclusively microliths, were made on Wommersom Quartzite (WSQ). While the above-cited raw materials have regionally occurring outcrops at distances of 20–50 km from the site, WSQ is an exogenous raw material that originates from outcrops situated at 115 km from the site.

**Table 4:** Siliceous raw materials employed for lithic productions in C12. When raw materials could not be determined, this was because of the small size of the artefacts or discolorations related to burning and patination.

	Direct Percussion		Microliths		Pressure	
Raw material	N	%	N	%	N	%
UTLT	232	41	12	20	16	44
UTMB	43	8	15	25	8	22
Wommersom	8	1	8	14	–	–
Fine-grained grey	110	19	7	12	6	17
Pebble-flint	36	6	5	8	–	–
Undetermined	135	24	12	20	6	17
Total	564	100	59	100	36	100

### The pressure bladelet production

**Fig 5:**
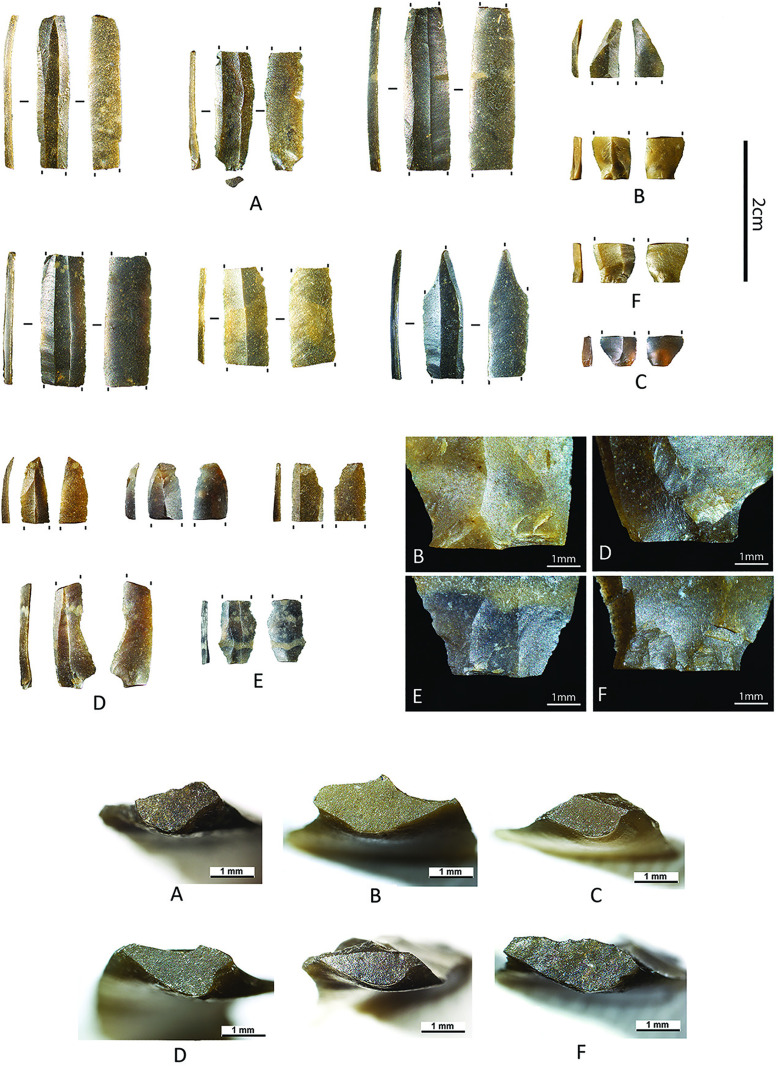
Pressure knapped bladelets from C12. The letters designate different views of the same artefact (e.g., for artefact B, we added a dorsal/profile photograph as well as a micrograph of the proximal part showing a summarily abraded overhang and a micrograph of the crescent-shaped butt).

**Fig 6:**
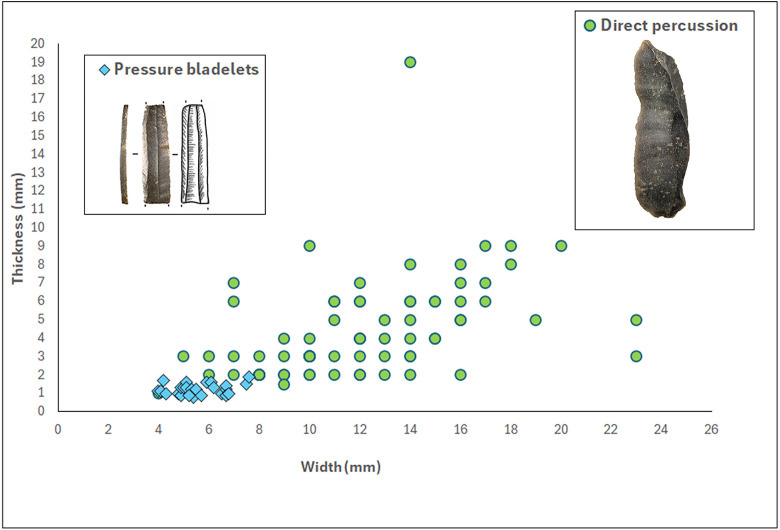
Width to thickness comparison of pressure knapped bladelets (n = 27) and the complete direct percussion bladelets (n = 58) of C12.

Apart from the above, C12 quite surprisingly yielded 36 microbladelet and microbladelet fragments that do not seem to fall within the metrical and techno-morphological range of bladelets that can be obtained through the use of direct percussion techniques ([34,35,38], Fig 5, S1 Table). The average width and thickness of these microbladelets (Fig 6) is respectively 5.4 mm and 1.4 mm, with a maximum width of 8.2 mm (and a median of 5.2 mm). Their original lengths are difficult to estimate due to the fact that nearly all of the bladelets are fragmented (n = 35), the longest fragment being only 22 mm long. Other than these metrical aspects, they seem to share several characteristics that allow to discriminate them from the other bladelets in C12. Generally speaking they have subparallel to parallel edges and dorsal ridges, with either trapezoidal or triangular sections depending on the occurrence of one to three dorsal ridges and in most cases, a relatively straight profile with a constant thickness from the proximal to the distal end. When the proximal ends are preserved (n = 12), in eight instances, a pronounced but short bulb is observed directly underneath the butt. Although being very small, the latter can vary considerably in shape, width and thickness (mean width and thickness of respectively 3.29 mm and 1.29 mm). Depending on the abrasion of the core-edge to reduce the overhang of previous removals, they can either be more or less elliptical in shape when abrasion is carried out (Fig 5E), or crescent-shaped when it is not (Fig 5B and 5D). The interior edge of the butts (at the intersection with the ventral side) is however always characterized by a smooth line, which contrasts greatly with the uneven interior edges observed on platforms obtained through direct percussion techniques [34]. Moreover, among the nine artefacts with preserved medial to distal ends, four display a slight distal curvature.

Combined, the above features indicate that this microbladelet assemblage was most likely produced by pressure knapping [5,39–43]. More precisely, to produce such narrow bladelets (width of 5 mm to 8 mm), the knappers in Kerkhove probably either applied pressure using a hand-held baguette (mode 1b) or by using a shoulder crutch (mode 2), according to the experimental research of J. Pelegrin [41,43, see also 38]. The use of indirect percussion can probably be dismissed in this case. The reduced size of the bladelets involved implies the use of small-sized cores that would have been difficult to keep inert while supporting the shock of a punch [5,9,42]. Because of this, it is highly doubtful that the indirect percussion technique allows the production of such microbladelets while maintaining a similar degree of regularity. At the same time, more experimental research is needed to verify how regular and how small bladelets produced through the indirect percussion technique can become (see discussion in [9]).

As opposed to the direct percussion bladelet *Chaîne Opératoire*, however, it is difficult to link any of the other artefacts in C12 to the pressure bladelet production. At first sight, based on their dimensions, the only possible candidates are the small backed bladelets (average widths of 4.91 mm and thickness of 1.79 mm), but again, assessing how these small backed bladelets were made and whether they could have been made on pressure knapped supports is not straightforward. Many of these microliths consist of small fragments that are sometimes intensively modified by backing and truncation, hampering the interpretation of the knapping technique and the identification of the type of blanks used to produce them. Only one partially backed bladelet seems to have been shaped on a blank with highly regular features (combining parallel edges and dorsal ridges, a trapezoidal section and a constant thickness).

**Fig 7:**
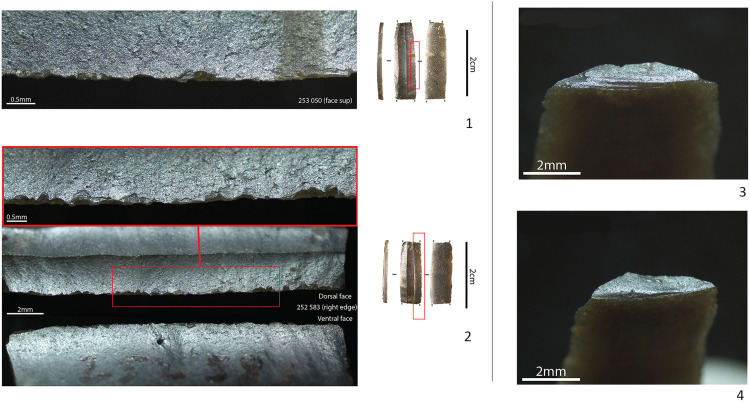
Use-wear and fracture types observed on the pressure bladelets. 1-2: Microscopic edge damage on the two longest pressure bladelets. For the bladelet 252 583, the more profound and darker scars are not related to use but linked to the sieving process, 3-4: Examples of the inverse bending fractures systematically observed on the pressure bladelets.

Furthermore, only a few functional observations could be made on the pressure bladelets. Incontestable use-wear traces are only present on the two longest and most regular medial bladelet fragments. They consist of bifacial and multi-directional microscopic edge-damage, more or less characteristic of repeated longitudinal contact with soft tissues, without however, evoking diagnostic impact damage (Fig 7: 1–2). Hence, while we cannot make far-reaching interpretations based on this quantitatively weak sample, it seems that the pressure bladelets could have served as lateral knife or dagger inserts (for cutting purposes) rather than as laterally hafted projectile implements. The lack of use-wear traces on the other bladelets suggests that they could have been considered as production waste. Selection perhaps focused on the most regular medial fragments, while leaving the proximal and distal fragments to be discarded in situ. The high fragmentation rate of the pressure bladelets (97% vs 81% of the direct percussion assemblage) could be a sign of an intentional breaking of the bladelets, to fit them in specific handles or slots. Analysis of the fractured surfaces reveals that nearly all are (inverse) bending fractures with weakly expressed *languettes* (Fig 7: 3–4). Yet, in spite of this rather systematic result, experimental research is again needed to verify whether this result could match the hypothesis of an intentional breaking or whether similar fractures could have also been obtained during debitage.

Finally, the pressure knapped bladelets are made on the same flint varieties as the direct percussion bladelets and the microliths of C12 (Table 4): primarily UTLT flint was employed for their production, followed by UTMB and fine-grained grey flint.

### The Refit analysis

**Fig 8:**
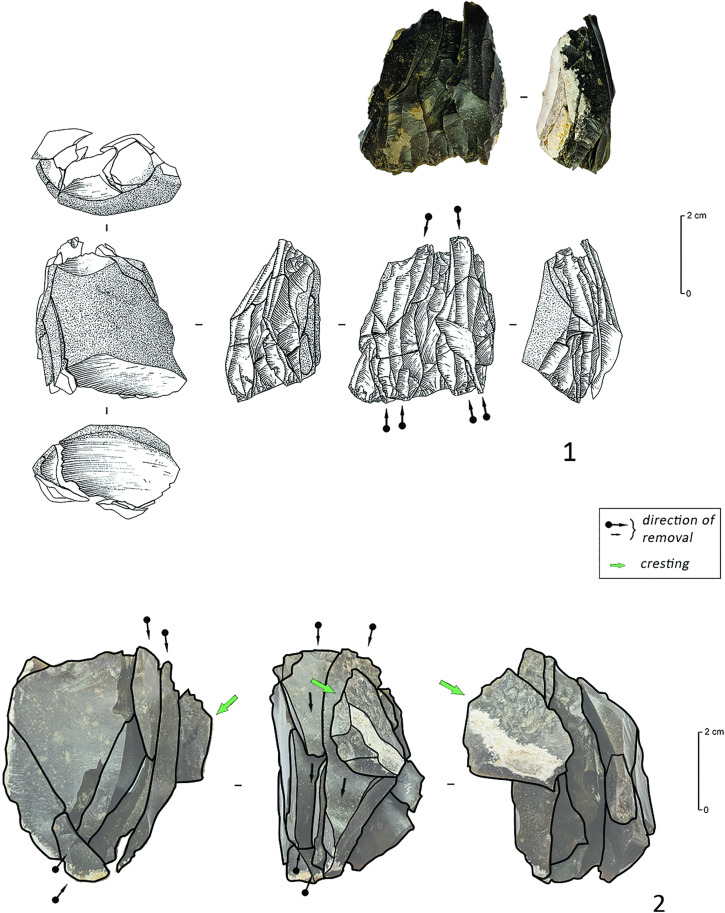
Direct percussion bladelet refit sequences from C12 (Drawings C. Delluc).

105 artefacts (17,62% of the artefacts > 1 cm) could be refitted, repartitioned over 31 refit sets. Although all the artefacts from C12 were included in the refitting analysis, only sequences related to the direct percussion *Chaîne Opératoire* could be reconstructed. The refits confirmed the first impressions provided by the attribute analysis. The two larger refit sets (R162, n = 12 and R164, n = 11; Fig 8) demonstrate that the striking platforms of the opposed platform cores were used in an alternating manner and one of the refit sets includes a transversally oriented removal, that can be interpreted as a remnant of the installation of a frontal crest. As such, these results are also in perfect agreement with earlier conclusions drawn from the refits of the other Middle Mesolithic clusters at the site [18].

**Fig 9:**
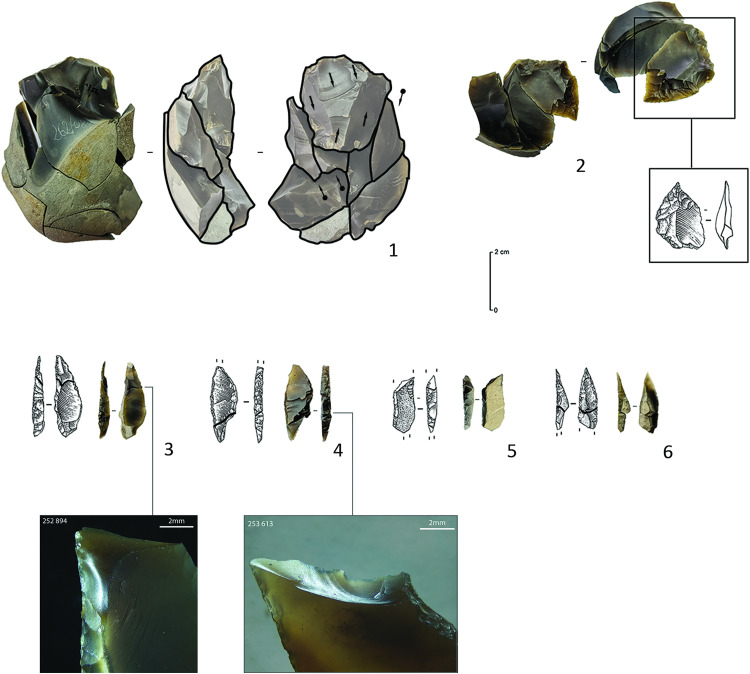
Refit sequences of the nodule of translucent grey flint with heavily rolled cortex. 1. R168; 2. Flake refits with rough-out of invasively retouched point; 3-6. Additional microliths and detailed views on the fracture surfaces of two of the microliths broken during retouch (in 3, a plunging retouch caused the fragmentation).

A third refit set (Fig 9: 1), R168 (n = 8), stands out compared to the previous two, as it represents a rather opportunistic flake debitage on a nodule of translucent grey flint with a heavily rolled cortex, that broke during debitage along pre-existing frost-fissures. Several additional short refit sets in all likelihood also resulted from this same knapping sequence (although they could not be refitted onto R168): the first is composed of five more or less rectangular flakes, one of which was intensively shaped by invasive retouch and seems to be a rough-out of an invasively retouched point (Fig 9: 2); The others consist of a group of four rather remarkable microliths (three backed points and a scalene triangle, Fig 9: 3–6). Three of these microliths broke during retouch, seemingly due to the highly variable thickness of – and the unusually large cortical remnants on – the bladelet supports chosen for their fabrication. Taken together, the debitage (aimed at flakes instead of bladelets) as well as the blanks selected for microlith production are therefore unusual and suggest that these sequences could have been the work of a less experienced knapper.

Finally, among the total amount of 105 artefacts that could be refitted, nine presented use-wear traces, mainly bearing witness to the fact that the tools derived from the direct percussion *Chaîne Opératoire* were engaged in an *ad hoc* manner, in the different activities cited above, i.e., butchery and hide-working activities, but probably also the processing of bones and plant materials.

## Discussion

### The homogeneity of C12

The refitting thus confirms that the artefacts in C12 are mainly related to the direct percussion *Chaîne Opératoire*. As opposed to this, none of the pressure knapped bladelets could be included in the refit sets (break refits nor ventral to dorsal refits could be made), despite many attempts and the complete recovery of the smallest artefact fraction during the excavation by sieving. By consequence, they seem to form an independent addition to the main debitage activities that were carried out in C12. From a functional point of view, their presence in the artefact cluster is best explained by the fact that they could have been produced at –or brought in to– the site as a part of a specific retooling activity. A scenario, for example, in which damaged lateral knife or dagger inserts were replaced by new specimens, whether or not intentionally broken to fit into slots, would correspond well with the few use-wear traces that were observed on the longer medial fragments. Such an interpretation would also explain the small quantity of pressure knapped bladelets, the presence of the many short proximal and distal fragments, as well as the total lack of refits.

The fact that the pressure bladelets consist of a complementary addition to the main debitage activities undertaken in C12, however, raises another important question. How certain can we be that the pressure bladelets are contemporaneous with the rest of the assemblage? And in other words, how certain are we that they date to the Middle Mesolithic?

Given the abundance of Early Mesolithic occupation traces at the site, an admixture in C12 of Early Mesolithic artefacts (and ecofacts, cf. the dated hazelnut shell) is to be expected. If such an admixture occurred, it must however have been a very slight admixture. The microlith composition, the spatial distribution of the microliths compared to that of the pressure bladelets as well as the raw materials used in both chaînes opératoires all argue in favor of the homogeneity of the artefact cluster and of a Middle Mesolithic date for the pressure bladelets. Only 6 out of 47 identifiable microliths (the triangles, backed points and point with retouched base) could potentially point to a residual Early Mesolithic presence in the cluster. However, they consist of the rather atypical specimens discussed above (cf. Fig 9: 3–6), potentially produced on the same fine-grained nodule as the unfinished point with invasive retouch. Some authors, in addition, believe that these specific microlith types continue to be in use during the first transitional stages of the Middle Mesolithic (for example in so-called “RMS-A” [21] and “Beuronien à lamelles à dos” assemblages [44]), meaning that in C12, the possibility that they have been produced by the same people that were responsible for the production of the small backed bladelets and invasively retouched points in the artefact cluster cannot be dismissed.

**Fig 10:**
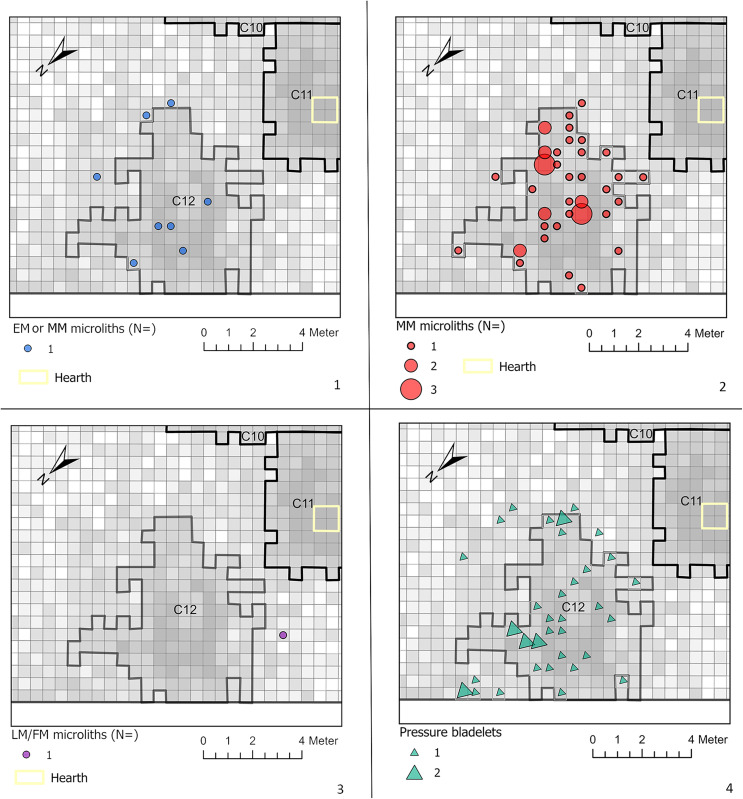
Spatial distribution of: 1. The Early or Middle (?) Mesolithic microliths, 2. The Middle Mesolithic microliths, 3. The Late/Final Mesolithic microliths and, 4. The pressure knapped bladelets in C12.

Secondly, the pressure knapped bladelets are spatially evenly dispersed across C12, coinciding with the equally evenly distributed Middle Mesolithic microliths in the cluster (Fig 10). They are not restricted to a specific sector or to the perimeter of the artefact cluster. And thirdly, as mentioned before, the raw materials used correspond well with the rest of the raw materials employed in C12. Based on these arguments, an Early Mesolithic age for the pressure bladelet assemblage seems unlikely.

The hypothesis of a Late Mesolithic age for the appearance of the pressure technique at the site can be rejected entirely. From the start of the Late Mesolithic onwards, paludal conditions and more particularly alder carr vegetation had extended over all but the highest parts in the alluvial plain [14], making the area less suitable for occupation. Although several isolated trapezes occur in the first excavation trench (hunting losses in this marshy landscape?), the only Late Mesolithic artefact cluster occurs at a distance of 200 m to the southwest, located higher and drier on the levee top. There, the bladelets moreover differ in the way they were produced (displaying similarities with the Essart A debitage method, [11]) as well as with regards to the raw materials employed.

### Interpreting the evidence of pressure knapping in NW Europe

**Fig 11:**
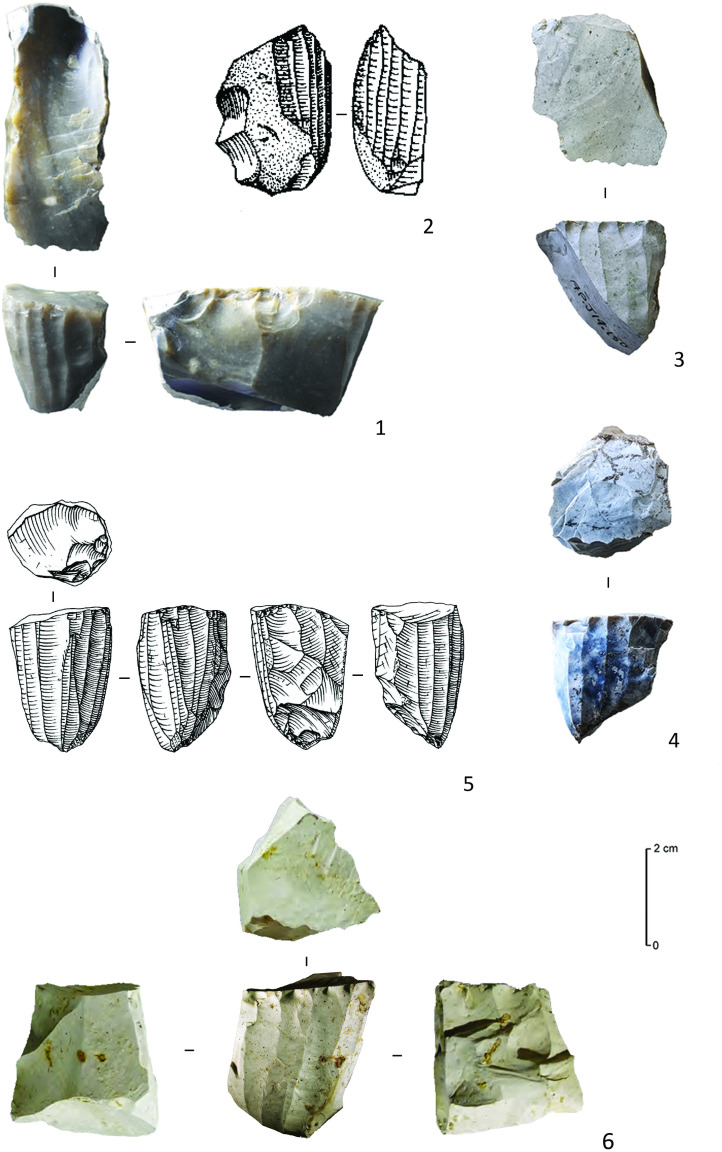
Example of pressure knapped cores. 1. Amiens rue Lecocq (FR, modified after [42], original photograph by S. Lancelot, INRAP); 2. Weelde – Paardsdrank (BE, modified after [45]; 3-4. Abri du Pape (BE); 5. Taarlo (NL, modified after [46]; 6.Champigny Mont-Saint-Pierre (FR).

Overall, the evidence available so far for the presence of pressure knapping in NW Europe is very scarce and consists of a series of cores that are identified as pressure knapped cores because of the regularity, narrowness and parallelism of the lamellar negatives on their debitage surface and because of their morphologies, sometimes resembling that of ‘handle cores’ (Fig 11; for a definition see [45]. Other characteristics are their overall limited sizes (which would preclude that they are the outcome of indirect percussion, see [9], the fact that they often lack overhang abrasion (resulting in a saw-toothed edge of the striking platform) and finally, a debitage surface that often displays a slight distal curvature. Examples of such cores have been found in Northern France at Choisy au Bac “La bouche d’Oise” [46], at the “Mont-Saint-Pierre” site in Champigny [47] and at the site of Amiens “rue Lecocq” [48,49]. Two other examples exist at the Belgian site of Abri du Pape in Anseremme [50, personal observation H. Vandendriessche]. Unfortunately, for the time being the contextual information and chronology of these sites is not straightforward to interpret. The former two are single finds in large-scale and chronologically diverse palimpsests. At Amiens, a test-pit yielded a typical handle core alongside of an elongated scalene triangle but also indirect percussion bladelets and a ^14^C-date at the beginning of the Late Mesolithic. The Abri du Pape, including its chronostratigraphic framework, is currently the subject of an extensive re-evaluation. Hence, the cores cannot be assigned to a specific Mesolithic occupation phase of the site yet. Besides the above-mentioned, based on drawings in older publications, a pressure knapped core could have also been found at Weelde-Paardsdrank [51] and at Dolembreux [20] although both need to be documented and analyzed in greater detail to confirm this.

Finds of pressure knapped cores in the Netherlands are to our knowledge restricted to two sites: Taarlo [52] and Leeuwarden “Hempens” [53,54] both situated in the northernmost part of the Netherlands. The find context of the core at Taarlo is not described and unfortunately, for the Hempens site, exact numbers and descriptions of the handle cores mentioned are lacking [53]. In Germany, evidence for pressure knapping seems almost completely absent in Nordrhein-Westfalen but becomes gradually more frequent towards the northern part of the country, in the provinces directly adjacent to and included in the Maglemosian core area, i.e., Niedersachsen and Schleswig-Holstein [55–59]. There, handle-cores are found in assemblages characterized either by elongated scalene triangles or trapezes and are respectively considered to date to the last part of the Early/Middle Mesolithic and the beginning of the Late Mesolithic, which seems confirmed by the few available ^14^C-dates [55–59].

Hence, the regular microbladelets from C12 constitute new proof for the use of pressure knapping in Northwestern Europe that can be added to this list. If we accept a Middle Mesolithic chronological position for the pressure bladelets, this means that pressure knapping was already present in the period dated between 9525–9212 and 8452–8224 cal. BP [23]. This is potentially almost as early as for example in the Late Maglemosian of Southern Scandinavia where pressure knapping occurs from ca. 9500 to 9200 cal BP onwards [3]. The occurrence of obliquely truncated small backed bladelets in C12 seems to lend further credibility to such an early appearance of pressure knapping at the site, due to the fact that they bear a striking resemblance to the elongated scalene triangles known from the Late Maglemosian, where they are also found together with pressure cores, for example at sites such as Ulkestrup II and Svaerdborg II [2,3].

Considering the above, we need to ask ourselves which mechanisms could have been responsible for this early introduction of pressure knapping in Northwest Europe. Three possibilities exist. Pressure knapping could have been a local invention, a so-called invention ‘ex nihilo’ (see [1]). An argument in favour of this could have been the fact that only some of the least technically complex forms of pressure knapping were attested, mode 1b and mode 2 [43]. However, this seems a bit far-fetched given the presence of pressure knapping, already in the course of the 10th and 9th millennium cal BP, in adjacent areas to the northeast, i.e., Southern Scandinavia [3] but also Northern Germany [56]. The more parsimonious explanation would be that either the movement of people (demic diffusion) from these areas or increased contacts with these areas could have been responsible for the knowledge transmission required to introduce pressure knapping in NW Europe.

Interestingly, the exact same question was asked concerning the initial spread of pressure knapping and “microblade technologies” from the east towards Fennoscandia, the Baltic States and finally also Southern Scandinavia. In this regard, Hartz et al. [56: 168] already proposed that microblade technology in the western Baltic was introduced there from the east following contacts with eastern Baltic hunter-gatherers, while Sörensen et al. [3: 28–29] concluded slightly later that the important shift in Scandinavian Mesolithic lithic technology that occurred with the introduction of pressure knapping in the course of the 10th millennium cal BP was the consequence of the fact that “*knowledge, and possibly people, of eastern and western origin came into contact and probably mixed in northern, central and southern Scandinavia and in the western Baltic area”* [3: 29]*.* This idea that pressure knapping could have been brought in by new people arriving from the east was subsequently corroborated by palaeogenetic evidence that confirmed the increasing presence of eastern hunter-gatherer (EHG) ancestry (with origins in the Upper Palaeolithic of Siberia [60: 2] in hunter-gatherer genomes throughout Scandinavia and the Baltic area from the 10th millennium cal BP onwards [60–62], with the exception of Denmark in which this presence of EHG ancestry appears to have remained relatively weakly expressed [63].

Should we interpret the appearance of pressure knapping in NW Europe as a (south)westward extension of similar population movements? The situation in NW Europe seems to have been of a different nature. In Scandinavia, the introduction of regular microblade productions is considered to have been a profound change in lithic technological organization. Pressure bladelets were systematically represented in lithic assemblages [2,59,64,65] and their production served specific purposes, e.g., they were produced, among others, as inserts for slotted bone points and daggers [66,67]. Moreover, in southern Scandinavia, pressure debitage exists from the Late Maglemosian (ca. 9500–9200 cal BP) to the beginning of the Ertebølle phase (ca. 7400 cal BP, cf [56]) and different pressure blade concepts are recognized throughout this period (conical core concept, handle-core concept/keeled core or wedge-shaped core [59,64,65]. In some regions of Scandinavia, as in Norway [65], the importance of pressure knapped microblades also clearly increased throughout the Mesolithic period.

**Fig 12:**
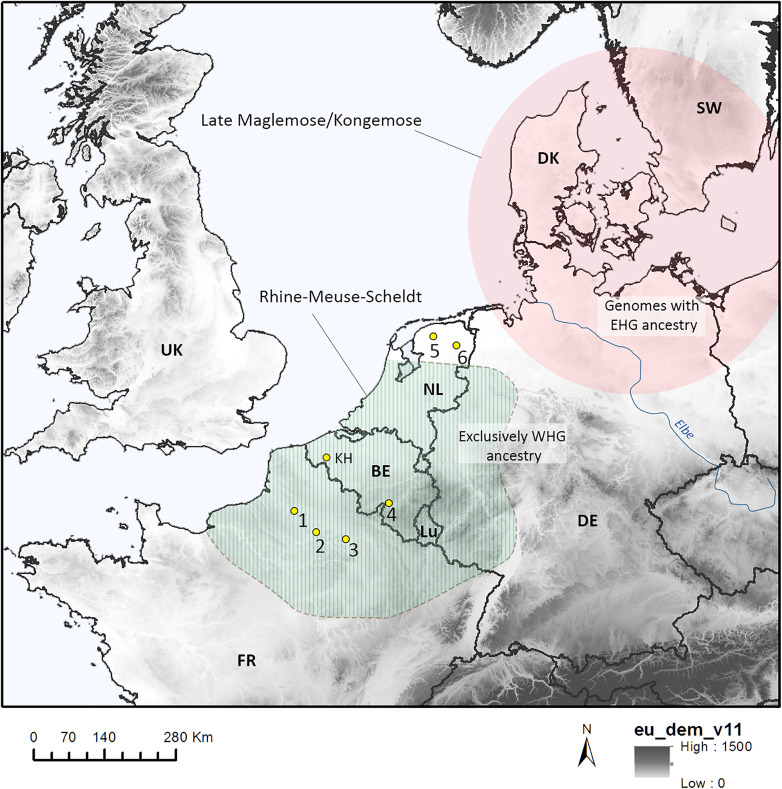
Sites in the Benelux and France with evidence for the use of pressure knapping and tentative boundaries of the RMS culture as well as the Maglemose/Kongemose culture. 1. Amiens- rue Lecocq; 2. Choisy au Bac – La Bouche d’Oise; 3. Champigny – « Mont Saint-Pierre »; 4. Abri du Pape; 5. Leeuwarden-Hempens; 6. Taarlo (data source for the DEM, https://doi.org/10.5270/ESA-c5d3d65).

This contrasts strongly with the paucity of the evidence we have in NW Europe. Apart from the Kerkhove bladelets, so far this evidence only consists of a handful of pressure cores. In addition, in theory, this evidence is potentially spread out over a period of ca. 2000 years, as the pressure cores are found in contexts that are often either linked with the Middle and/or Late Mesolithic. Compared to the Scandinavian case, this seems to argue against substantial demic diffusion being at the origins of pressure knapping in NW Europe. Perhaps pre-existing social/territorial boundaries could have simply limited such population movements and the wider-scale adoption of pressure knapping? Indeed, during the 10^th^ and 9^th^ millennium cal BP, Northwestern Europe is considered to have been part of the so-called Beuronian cultural sphere [68,69] and the RMS culture [21]. These boundaries could have been permeable to some extent, for the dispersal of small groups of people and ideas, but could have prevented more generalized population flows and hence a more generalized adoption of the technique and tools linked to it. This scenario again agrees rather well with the genetic evidence that is available for the moment. EHG-ancestry remains entirely absent west of the Elbe, where Mesolithic hunter-gatherer genomes are exclusively characterized by so-called western hunter-gatherer ancestry (WHG) [62,70]. This divide corresponds to a large extent with the contact zone between the spheres of influence of the RMS culture and the Maglemosian (Fig 12).

Finally, we need to take into account that the overview portrayed above is to a certain extent affected by the state of the research. The small-sized bladelets and cores presented in this paper could have easily been overlooked in studies of Middle and Late Mesolithic assemblages in the past, especially if they also fulfilled a secondary role in lithic productions on other sites as it seems to have been the case in Kerkhove. Their discovery could have also been hindered by a lack of adapted excavation strategies. Wet-sieving on small enough mesh sizes is a prerequisite to recover the smaller debitage elements described above. In addition, we surmised that the pressure bladelets at the site could have served to equip highly specific tool types, e.g., akin to the slotted bone points and daggers that are common in the Maglemosian/Kongemosian area and in the Baltic. Insufficient attention has probably been paid to the occurrence of pressure microbladelets related to these kinds of tools due to the fact that such organic implements have not been documented yet in NW Europe, with the exception of a slotted bone point from the area of Wiesbaden, discovered in the early 1800s [71]. More detailed technological research is by consequence needed to shed more light on this matter and to confirm whether the current status of pressure bladelet productions in NW Europe could have corresponded to a historical reality.

## Conclusion

The technological and functional research carried out on C12 contributes important new data to our understanding of the application of pressure knapping in Northwestern Europe during the Mesolithic. Most importantly, it indicates an early emergence of this technique, from the Middle Mesolithic onwards, which in turn indicates that the knowledge responsible for this in all likelihood originated from the Maglemosian of Northern Germany/Southern Scandinavia. At the same time, this discovery raises a few important questions. If already present at this time, why does the evidence for its use remain so scarce throughout the Mesolithic and why does the situation in Northwestern Europe contrast so strongly with the Scandinavian and Baltic case, where pressure knapping rapidly evolved to become an important component of lithic technological organisation after its introduction? Genetic evidence points to an introduction of this technique in the latter areas, at least in part by demic diffusion from the east and concomitantly seems to indicate the absence of such population flows towards NW Europe, perhaps hindered by existing social boundaries. Having said this, it is not clear for the moment to which extent the scarcity of the evidence in NW Europe reflects a historical reality or whether this image is created by a research bias and could still change substantially in the years to come. Renewed technological research, specifically on Middle and Late Mesolithic sites will play a crucial role either in confirming or refuting the hypotheses outlined above. The same holds true with regard to the genetic evidence. Although for the time being, admixture of EHG ancestry is non-existent in western Europe, this is based on a relatively restricted sample of reconstructed genomes. In other words, future research might yield new data for the 9th and 8th millennium cal BP that could create a more diversified picture. To conclude, the discovery of pressure knapped bladelets in the Middle Mesolithic of Kerkhove, amidst a direct percussion bladelet assemblage and far away from Fennoscandia and the Baltic region should perhaps also incite us to re-think the often too rigid dichotomous vision we have in Northwestern Europe of an Early/Middle Mesolithic period solely characterized by direct percussion productions compared to a Late Mesolithic solely characterized by indirect percussion assemblages.

## Supporting information

S1 Table Principal technological characteristics of the pressure bladelets found in C12. (XLSX)

## References

[1] Inizan M.-L. Pressure débitage in the Old World: Forerunners, researchers, geopolitics–handing on the baton. In: Desrosiers P, editor. The emergence of pressure blade making: From origin to modern experimentation. Boston: Springer; 2012. p. 11–42.

[2] Sørensen M. The arrival and development of pressure blade technology in Southern Scandinavia. In: Desrosiers P, editor. The emergence of pressure blade making: From origin to modern experimentation. Boston: Springer; 2012. p. 237–59.

[3] Sørensen M, Rankama T, Kankaanpää J, Knutsson K, Knutsson H, Melvold S, et al. The First Eastern Migrations of People and Knowledge into Scandinavia: Evidence from Studies of Mesolithic Technology, 9th-8th Millennium BC. Norwegian Archaeol Rev. 2013;46(1):19–56. doi: 10.1080/00293652.2013.770416

[4] Biagi P, Starnini E. The origin and spread of the Late Mesolithic Blade and Trapeze Industries in Europe: reconsidering JGD Clark’s hypothesis fifty years after. In: Terna S, Govedarica B, editors. Interactions, changes and meanings. Essays in honour of Igor Manzura on the occasion of his 60th birthday. Kishinev: Kishinev University; 2016. p. 33–45.

[5] Binder D, Collina C, Guilbert R, Perrin T, Garcia-Puchol O. Pressure-knapping blade production in the North-Western Mediterranean Region during the seventh millennium cal BC. In: Desrosiers P, editor. The emergence of pressure blade making: From origin to modern experimentation. Boston: Springer; 2012. p. 199–217.

[6] Marchand G, Perrin T. Why this revolution? Explaining the major technical shift in Southwestern Europe during the 7th millennium cal. BC. Quat Int. 2017;428:73–85. doi: 10.1016/j.quaint.2015.07.059

[7] Perrin T, Dachy T, López-Montalvo E, Manen C, Marchand G. Quelles relations entre l’Afrique du Nord et l’Europe au début de l’Holocène? Tabona. Revista de Prehistoria y de Arqueología. 2022; 22:13–34.

[8] Rozoy J-G. L’étude du matériel brut et des microburins dans l’Epipaléolithique (Mésolithique) franco-belge. Bulletin de la Société préhistorique française. 1968;65(1):365–90. doi: 10.3406/bspf.1968.4157

[9] Allard P. Variability of laminar debitage in the second Mesolithic and early Neolithic in the north of France (7th and 6th millennium BCE). J Lithic Stud. 2017;4(2):75–103.

[10] Marchand G. Eléments pour la définition du Retzien. In: Thévenin A, Bintz P, editors. L’Europe des derniers chasseurs: Epipaléolithique et Mésolithique. Actes du Colloque international UISPP. Commission XII 5 Septembre 18-23. Grenoble, Paris: CTHS; 1995. p. 213–24.

[11] Messiaen L, Vandendriessche H, Crombé P. The Neolithization Process in the Lower-Scheldt Basin (Belgium, mid-6th to mid-4th Millennium cal BC) from a Lithic Technological Perspective. Lithic Technol. 2022;48(2):168–93. doi: 10.1080/01977261.2022.2109354

[12] Pironneau C, Aluwé K, Crombé P, Vandendriessche H. L’exploitation des mammifères au cours du Mésolithique dans le nord de la Belgique: le cas des occupations du Mésolithique ancien de Kerkhove (vallée de l’Escaut). Bulletin de la Société préhistorique française. 2023;120(4):603–20.

[13] Crombé P, Storme A, Cruz F, Allemeersch L, Vandendriessche H, Deforce K, et al. Early Holocene slope erosion in the Scheldt basin (Belgium): Naturally and/or human induced? Geomorphology. 2019;337:79–93. doi: 10.1016/j.geomorph.2019.03.025

[14] Cruz F, Sergant J, Storme A, Allemeersch L, Aluwé K, Jacops J, et al. Méthodologie d’une recherche paléoenvironnementale en archéologie préventive. L’exemple de Kerkhove-Stuw. In: Déak J, Ampe C, Mikkelsen J, editors. Soils as records of past and present. From soil surveys to archaeological sites: research strategies for interpreting soil characteristics. Proceedings of the Geoarchaeological Meeting Bruges, 6 & 7 November 2019. Bruges: Raakvlak; 2019. p. 175–88.

[15] Cruz F, Storme A, Allemeersch L, Sergant J, Vandendriessche H, Aluwé K, et al. Le paléoenvironnement de l’Escaut moyen sur le site de Kerkhove Stuw (Flandre Occidentale, Belgique) au cours de l’Holocène inférieure. Géomorphol Relief Proces Environ. 2021; 27(4):243–62.

[16] Vandendriessche H, Guéret C, Aluwé K, Messiaen L, Cruz F, Storme A, et al. Deux millénaires d’occupations mésolithiques au bord de l’Escaut à Kerkhove (Belgique) : première approche palethnographique. Bulletin de la Société préhistorique française. 2019;116(2):283–316. doi: 10.3406/bspf.2019.15001

[17] Vandendriessche H, Crombé P. Formalized Reduction Sequences from the Site of Kerkhove, Belgium – New Perspectives on Early Mesolithic Flint Knapping. Lithic Technol. 2020;45(2):110–24. doi: 10.1080/01977261.2020.1721162

[18] Vandendriessche H. Flintknapping from the Lateglacial to the Early Holocene: the Belgian Scheldt valley sites of Ruien and Kerkhove. Leiden: Sidestone Press; 2022.

[19] Vandendriessche H, Van Maldegem E, Crombé P. Catching a glimpse of mesolithic settlement patterns and site re-occupation through lithic refitting, raw material characterizations and absolute dating. J Archaeol Method Theory. 2023;30(1):239–67.

[20] Gob A. Les industries microlithiques dans la partie sud de la Belgique. In: Cahen D, Haesaerts P, editors. Peuples chasseurs de la Belgique préhistorique dans leur cadre naturel. Bruxelles: Patrimoine de l’institut Royal des sciences naturelles de Belgique; 1984. p. 195–210.

[21] Gob A. Extension géographique et chronologique de la culture Rhein-Meuse-Schelde (RMS). Helinium. 2023;25(1):23–36.

[22] Vermeersch P. Du Paléolithique final au Mésolithique dans le Nord de la Belgique. In: Cahen D, Haesaerts P, editors. Peuples chasseurs de la Belgique préhistorique dans leur cadre naturel. Bruxelles: Patrimoine de l’institut Royal des sciences naturelles de Belgique; 1984. p. 181–93.

[23] Crombé P. Mesolithic projectile variability along the southern North Sea basin (NW Europe): Hunter-gatherer responses to repeated climate change at the beginning of the Holocene. PLoS One. 2019;14(7):e0219094. doi: 10.1371/journal.pone.0219094 31314774 PMC6636730

[24] Robinson E, Van Strydonck M, Gelorini V, Crombé P. Radiocarbon chronology and the correlation of hunter–gatherer sociocultural change with abrupt palaeoclimate change: the Middle Mesolithic in the Rhine–Meuse–Scheldt area of northwest Europe. J Archaeol Sci. 2013;40(1):755–63. doi: 10.1016/j.jas.2012.08.018

[25] Crombé P, Storme A, Perdaen Y, Vandendriessche H. The changing role of hazelnuts (Corylus avellana) in the Mesolithic diet: The Scheldt basin (W Belgium) as a case-study. Quat Sci Rev. 2023;317:108295. doi: 10.1016/j.quascirev.2023.108295

[26] Cahen D. Das Zusammensetzen geschlagener Steinartefakte. Archäologisches Korrespondenzblatt. 1976;6(2):81–93.

[27] Cahen D, Karlin C, Keeley LH, Van Noten F. Méthodes d’analyse technique, spatiale et fonctionnelle d’ensembles lithiques. Helinium. 1980;20(3):209–59.

[28] Cziesla E. On refitting of stone artefacts. In: Cziesla E, Eickhoff S, Arts N, Winter D, editors. The Big Puzzle. International Symposium on Refitting Stone Artefacts, Monrepos 1987. Bonn: Holos; 1990. p. 9–44.

[29] De Bie M. Benefiting from refitting in intra-site analysis: lessons from Rekem (Belgium). In: Schurmans U, De Bie M, editors. Fitting rocks: Lithic refitting examined (British Archaeology Reports international series, 159). Oxford: Archaeopress; 2007. p. 31–44.

[30] De Bie M, Caspar JP. Rekem. A Federmesser Camp on the Meuse River Bank (Archeologie in Vlaanderen 3/Acta Archaeologica Lovaniensia 10). Asse-Zellik/Leuven: IAP/Leuven University Press; 2000.

[31] Cahen D, Moeyersons J. Subsurface movements of stone artefacts and their implications for the prehistory of Central Africa. Nature. 1977;266(5605):812–5. doi: 10.1038/266812a0

[32] Morrow TM. Lithic refitting and archaeological site formation processes: A case study from the Twin Ditch site, Greene County, Illinois. In: Odell GH, editor. Stone tools: Theoretical insights into human prehistory. Boston, MA: Springer US; 1996. p. 345–73.

[33] Villa P. Conjoinable Pieces and Site Formation Processes. Am Antiq. 1982;47(2):276–90. doi: 10.2307/279901

[34] Pelegrin J. Les techniques de débitage laminaire au Tardiglaciaire: critères de diagnose et quelques réflexions. In: Valentin B, Bodu P, Christiansen M, editors. L’Europe centrale et septentrionale au Tardiglaciaire, actes de la table ronde internationale (Nemours, 1997). Nemours: APRAIF (Mémoires du musée de Préhistoire d’Ile-de-France); 2000. p. 73–86.

[35] Sorensen M. Rethinking the lithic blade definition: towards a dynamic understanding. In: Apel J, Knutsson K, editors. Skilled production and social reproduction: aspects of tradional stone-tool technologies: proceedings of a symposium in Uppsala (Uppsala, 2003) (Stone Studies, 2). Uppsala: SAU; 2006. p. 277–98.

[36] Damlien H. Striking a difference? The effect of knapping techniques on blade attributes. J Archaeol Sci. 2015;63:122–35. doi: 10.1016/j.jas.2015.08.020

[37] Vandendriessche H, Crombé P, Collin JP. The cretaceous outcrops of the Lille-Tournai (FR/BE) area and their archaeological significance. Notae Praehistoricae. 2021;41:121–31.

[38] Radinović M, Kajtez I. Outlining the knapping techniques: Assessment of the shape and regularity of prismatic blades using elliptic Fourier analysis. J Archaeol Sci Rep. 2021;38:103079. doi: 10.1016/j.jasrep.2021.103079

[39] Tixier J. Le debitage par pression. In: Tixier J, Inizan ML, Roche H, editors. Préhistoire de la Pierre Taillée, t. 2 : Économie du débitage laminaire, IIIème table-ronde de technologie lithique, Meudon-Bellevue, octobre 1982. Antibes: CREP; 1984. p. 57–70.

[40] Flenniken JJ. The Paleolithic Dyuktai pressure blade technique of Siberia. Arctic Anthropol. 1987:117–32.

[41] Pelegrin J. Débitage expérimental par pression: « du plus petit au plus grand ». Technologie préhistorique. 1988; 25: 55–62.

[42] Pelegrin J. Long blade technology in the Old World: an experimental approach and some archaeological results. In: Apel J, Knutsson K, editors. Skilled Production and Social Reproduction, Aspects of Traditional Stone-Tool Technologies, Proceedings of a Symposium in Uppsala, August 20–24, 2003 (Stone Studies, 2). Uppsala: Societas Archeologica Upsaliensis; 2006. p. 37–68.

[43] Pelegrin J. New experimental observations for the characterization of pressure blade production techniques. In: The emergence of pressure blade making: From origin to modern experimentation. Boston, MA: Springer US; 2012. p. 465–500.

[44] Ducrocq T. Élements de chronologie absolue du Mésolithique dans le Nord de la France. In: Crombé P, Van Strydonck M, Sergant J, Boudin M, Bats M editors. Chronology and Evolution within the Mesolithic of North-West Europe: Proceedings of the International Congress Chronology and Evolution in the Mesolithic of Northwest Europe (May 30th till June 1st 2007, Brussels). Cambridge: Cambridge Scholar Publishing. p. 95–112.

[45] Söderlind S. The Handle Core Concept: Lithic Technology and Knowledge Transmission. Sidestone Press; 2024.

[46] Guéret C, Pelegrin J, Valentin B. Révision taphonomiques à propos du Mésolithique moyen et récent à la « Bouche d’Oise » à Choisy-au-Bac (Oise). In: Valentin B, editor. Paléolithique final et Mésolithique dans le Bassin parisien et ses marges, CNRS-UMR 7041, Projet collectif de recherche. Rapport d’activités pour 2009. Nanterre Cedex: Equipe d’ethnologie préhistorique; 2009. p. 263–74.

[47] Garmond N, Bouquin D, Guéret C, Poupon F, Toulemonde F. Du mésolithique en creux. Sépulture, fosses et mobilier sur le site du «mont saint-pierre» à champigny (marne), IXe-VIIe millénaires avant notre ère. Revue archéologique de l’Est. 2022:31–55.

[48] Ducrocq T. Découverte d’un nucléus à lamelles de type nordique « handle core – nucléus à poignée » dans le Mésolithique du nord de la France. Bulletin de la Société préhistorique française. 2021;118(2):392–5.

[49] Ducrocq T. Le Mésolithique – dynamique de peuplement à l’échelle de la région. In: Jagerschmidt-Seguin A, Paris C, editors. La Somme des Préhistoires. Gand: Snoeck; 2024. p. 195–208.

[50] Léotard JM, Straus LG, Otte M. L’abri du Pape. Bivouacs, enterrements et cachettes sur la Haute Meuse belge: du Mésolithique au Bas Empire Romain (ERAUL 88). Liège: Presses universitaires de Liège;1999. p. 352.

[51] Huyge D, Vermeersch PM. Late Mesolithic settlement at Weelde-Paardsdrank. In: Vermeersch PM, editor. Contributions to the study of the Mesolithic of the Belgian Lowland (Studia Praehistorica Belgica I). 1982. p. 115–203.

[52] Musch J, Peeters H. Signalement: Klingproductie met behulp van de druktechniek in het Nederlandse neolithicum? Archeologie. 1993;4:83–91.

[53] Noens G. Een afgedekt mesolithisch nederzettingsterrein te Hempens/N31 (gemeente Leeuwarden, provincie Friesland, Nl.). Vol. 7, Archaeological reports Ghent University (ARGU). Gent: Academia Press; 2011. p. 288.

[54] Peeters JHM, Raemaekers DCM, Devriendt IIJALM, Hoebe PW, Niekus MJLTh, Nobles GR et al. Paradise lost? Insights into the early prehistory of the Netherlands from development-led archaeology (NAR 62). Amersfoort: Cultural Heritage Agency of the Netherlands; 2017. p. 259.

[55] Gerken K. Studien zur jung-und spätpaläolithischen sowie mesolithischen Besiedlung im Gebiet zwischen Wümme und Oste (Archäologische Berichte des Landkreises Rotenburg 9). Oldenburg: Isensee verlag; 2001. p. 361.

[56] Hartz S, Terberger T, Zhilin M. New AMS-dates for the Upper Volga Mesolithic and the origin of microblade technology in Europe: Neue AMS-Daten zum Mesolithikum der oberen Wolga und das Aufkommen der Mikroklingentechnik in Europa. Quartär–Internationales Jahrbuch zur Erforschung des Eiszeitalters und der Steinzeit. 2010;57:155–69.

[57] Söderlind S. A study of the mesolithic handle core technology in Schleswig-Holstein. Archäol Inf. 2018;41:305–16.

[58] Lübke H. Spät- und Endmesolithische Kustensiedlungsplätze in der Wismarbucht. Neue Grabungsergebnisse zur Chronologie und Siedlungsweise. Bodendenkmalpflege in Mecklenburg-Vorpommern. 2004; 52: 83–110.

[59] Söderlind S, Serbe B, Groß D. I would walk 500 miles… Mesolithic handle core networks in the western Baltic region. In: Stones. Current stone-age research in Northern Europe. Uppsala: Uppsala Universitet; 2023. p. 178–91.

[60] Mittnik A, Wang C-C, Pfrengle S, Daubaras M, Zariņa G, Hallgren F, et al. The genetic prehistory of the Baltic Sea region. Nat Commun. 2018;9(1):442. doi: 10.1038/s41467-018-02825-9 29382937 PMC5789860

[61] Günther T, Malmström H, Svensson EM, Omrak A, Sánchez-Quinto F, Kılınç GM, et al. Population genomics of Mesolithic Scandinavia: Investigating early postglacial migration routes and high-latitude adaptation. PLoS Biol. 2018;16(1):e2003703. doi: 10.1371/journal.pbio.2003703 29315301 PMC5760011

[62] Posth C, Yu H, Ghalichi A, Rougier H, Crevecoeur I, Huang Y, et al. Palaeogenomics of Upper Palaeolithic to Neolithic European hunter-gatherers. Nature. 2023;615(7950):117–26. doi: 10.1038/s41586-023-05726-0 36859578 PMC9977688

[63] Allentoft ME, Sikora M, Refoyo-Martínez A, Irving-Pease EK, Fischer A, Barrie W, et al. Population genomics of post-glacial western Eurasia. Nature. 2024;625(7994):301–11. doi: 10.1038/s41586-023-06865-0 38200295 PMC10781627

[64] Sørensen M. Rethinking the lithic blade definition: towards a dynamic understanding. In: Apel J, Knutsson K, editors. Skilled Production and Social Reproduction, Aspects of Traditional Stone-Tool Technologies, Proceedings of a Symposium in Uppsala, August 20–24, 2003 (Stone Studies, 2). Uppsala: Societas Archeologica Upsaliensis; 2006. p. 277–96.

[65] Damlien H, Nyland AJ, Redmond JJ. Lithic technology before and after the Storegga tsunami (8200 cal BP): Dissolving large-scale regional trends to identify social impact of crisis in western Norway. Holocene. 2024;34(12):1790–806. doi: 10.1177/09596836241274987

[66] Bokelmann K. Zum Beginn des Spätmesolithikums in Südskandinavien. Geweihaxt, Dreieck und Trapez, 6100 cal BC. Offa. 1999;56:183–97.

[67] Sørensen SA. The Kongemose Culture. Syddansk Universitetsforlag; 2017.

[68] Taute W. Neue Forschungen zur Chronologie von Spätpaläolithikum und Mesolithikum in Süddeutschland. Archäologische Informationen. 1973;2–3:59–66.

[69] Kozlowski SK. Thinking Mesolithic. Oxford: Oxbow Books. p. 545.

[70] Rivollat M, Deguilloux MF. Diversité génomique des groupes mésolithiques en Europe: origine, évolution et métissages. Bulletin de la société archéologique champenoise. 2020;113(2–3):375–94.

[71] Manninen MA, Asheichyk V, Jonuks T, Kriiska A, Osipowicz G, Sorokin AN, et al. Using Radiocarbon Dates and Tool Design Principles to Assess the Role of Composite Slotted Bone Tool Technology at the Intersection of Adaptation and Culture-History. J Archaeol Method Theory. 2021;28(3):845–70. doi: 10.1007/s10816-021-09517-7

